# Infants infer third-party social dominance relationships based on visual access to intergroup conflict

**DOI:** 10.1038/s41598-022-22640-z

**Published:** 2022-10-29

**Authors:** Anthea Pun, Susan A. J. Birch, Andrew Scott Baron

**Affiliations:** University of British Columbia, Vancouver, Canada

**Keywords:** Evolution, Psychology

## Abstract

During a conflict, having a greater number of allies than the opposition can improve one’s success in a conflict. However, allies must be aware that has a conflict has occurred, and this is often influenced by what they are able to see. Here, we explored whether infants’ assessment of social dominance is influenced by whether or not social allies have visual access to an episode of intergroup conflict. In Experiment 1, 9–12-month-olds only expected an agent to be socially dominant *if* their allies were able to witness the conflict. Experiment 2 provided further support for this finding, as infants did *not* expect an agent from a numerically larger group to be socially dominant when allies were *unable to witness* the conflict. Together, these results suggest that infants do not simply use a heuristic in which “numerically larger groups are always more dominant”. Importantly, infants are able to incorporate social allies’ ability to witness a conflict when predicting social dominance between groups.

## Introduction

Social dominance based on relative numerical group size is a critical cue that helps various social species assess their risk of engaging in a conflict. Indeed, observations of non-human primates, lions, hyenas and even insects have revealed that attacks against outgroup members are more likely to occur when opponents are outnumbered, or when individuals stray from their allies^1,2^. This suggests that when facing a conflict, individuals can gain “strength” in numbers when allied with others, and numerically larger groups are often perceived to be socially dominant over numerically smaller groups^1–3^. However, it is important to emphasize that social dominance based on relative numerical group size is a *derived* form of dominance^4^, which changes across time and contexts (i.e., not a stable trait). For example, even if individuals have many social allies within their community, not all allies may be present or bear witness to a conflict. And, individuals often make decisions about how they should behave (e.g. retrieving food, defending territory), and how others are likely to behave towards them (i.e., will I be attacked, and how likely am I to win?) based on the present circumstances^5^. Therefore, it is critical to keep track of whom is watching, and whether they can witness one’s actions. The present study explores whether infants incorporate the perspectives of multiple agents when deriving social dominance between groups, or whether their judgments are predicated solely on a mathematical assessment of numerical majority.

Data from studies with non-human animals underscores the importance of attending to others’ eyes during social reasoning^6,7^. Indeed, interpreting what others know based on their eye cues (eg. eye gaze, joint attention) is essential when coordinating non-verbal collaborative interactions such as hunting, gathering and soliciting social allies^7–13^.

However, the developmental origins of such “mind reading” based on visual access requires further investigation. Some developmental work has revealed that human infants understand that an agents’ presence or absence may affect their knowledge and behavioral expectations^14–17^. For example, after a friendship had been established between agent A and B, 13 month- old infants saw agent B hit another agent, C. When A was present to witness B’s harmful actions, infants expected A to alter their behavior and avoid B. In contrast, when A was absent and did not witness B’s transgression, infants expected A to continue their friendship with B^14^. Similarly, 1- year old infants’ expectation of third-party punishment was influenced by whether a bystander was present, or absent during a wrongdoer’s transgression towards the bystanders’ ingroup member. When the bystander was present, infants expected the bystander to punish the wrongdoer. In contrast, when the bystander was absent and did not witness the transgression against their ingroup member, infants did not expect the bystander to punish the wrongdoer^17^. Together, these results suggest that infants within the first year of life appear to understand that an agents’ knowledge and subsequent behavior is influenced by what they do, or do not witness.

Indeed, humans’ preferential attention to eyes (from birth) may help facilitate the ability to interpret and predict others’ behavior^7^. However, the aforementioned studies cannot speak directly to whether infants understand that what an agent sees specifically through the *eyes* is critical for reasoning about others’ perspectives. Furthermore, nothing is currently known about infants’ capacity to infer the knowledge of *social groups* based specifically through others’ eyes, and what they can see. Whereas previous work has focused on infants’ ability to use eye gaze to track objects or an individual’s behavior^13,18,19^, the current research explores for the first time how visual access influences infants’ third-party evaluations of multiple agents that belong to social groups.

The present study examined whether infants’ predictions about social dominance is directly influenced by whether social allies *see* (or do not see) intergroup conflict occur. Because infants within the first year of life expect a group with *more* social allies to be dominant^3^ and also understand that barriers can obstruct what one is able to see^20,21^, we reasoned that only the group that can *see* the conflict should be incorporated into infants’ predictions of social dominance (regardless of the *overall* group size). However, if judgments of social dominance are derived solely through mathematical comparison, then infants should expect the majority group to be dominant (regardless of whether social group members are able to witness the conflict or not).

To test this hypothesis, we conducted two experiments. In Experiment 1, 9–12 month old infants were introduced to two groups that were identical in numerical and physical size (3 agents in each group). When two identically sized agents from opposing groups attempted to cross a platform at the same time, a conflict occurred. Importantly, only one group could witness the conflict, as the other groups’ eyes were obstructed by a barrier. Although overall group size was equated, we hypothesized that only the agent whose social allies were able to view the conflict would be expected to prevail, and be socially dominant. In Experiment 2, we introduced 9–12-month old infants to two groups, differing in numerical size. One group had more members than the other (3 agents vs. 2 agents) (see^3^). However, in this current experiment, *no* social allies were able to witness the conflict, as their eyes were obstructed by barriers. This resulted in two identically sized agents engaged in a conflict while their respective allies remained unaware of the ensuing conflict. Consequently, we hypothesized that the agent with more allies present (but unable to see the conflict) would be no more likely to be dominant than the agent with fewer allies. What ultimately matters is who has more allies ‘in the know’. However, if infants instead rely on simple numerical majority when computing social dominance, they should expect the agent from the numerically larger group to prevail, and be socially dominant.

## Experiment 1

### Method

#### Ethics approval

The University of British Columbia and the Behavioral Research Ethics Board approved all methods and experimental protocols (approval no. H10-00147). All research methods and experimental protocols described in this manuscript were conducted in accordance with the University of British Columbia’s Behavioral Research Ethics Board guidelines and regulations (approval no. H10-00147). Since our participants were infants, a legal guardian provided informed consent on behalf of each participant.

#### Participants

For Experiment 1, data from 48 infants (mean age = 10.54 mo, range = 9.00 mo–12.00 mo, SD = 26 d, 25 females) were recruited from a university database and informed consent from each participants’ legal guardian was provided. Reports from the legal guardian indicated that our final sample was composed of 42% Caucasian, 34% East Asian and 24% that identified as other ethnicity/ethnicities. Consistent with the inclusion criteria implemented by Pun and colleagues^3^ and Thomsen and colleagues^23^, an additional 11 participants were excluded from the sample because they did not watch the screen during the critical sequence in which one of the agents bowed down and moved out of the other agent’s path of motion (n = 5), fussed out (n = 5), or because of sibling or parental interference (n = 1).

#### Procedure

The University of British Columbia and the Behavioral Research Ethics Board approved all methods and experimental protocols (approval no. H10-00147). All research methods and experimental protocols described in this manuscript were conducted in accordance with the University of British Columbia’s Behavioral Research Ethics Board guidelines and regulations (approval no. H10-00147). For both experiments, the procedure was identical. As in the procedure implemented by Pun and colleagues^3,22^, each participant was seated on the lap of their caregiver in a sound proof testing room for the duration of the experiment, ∼140 cm from the center of a television screen measuring 48″ in diameter. To ensure that caregivers would not influence their child’s behavior, they were instructed to either keep their eyes closed or were asked to wear a pair of opaque glasses. Caregivers were also asked to remain silent and not direct the child’s’ attention. The experimenter sat adjacent to the infant and caregiver, separated by a distance of ∼4 feet and hidden behind a black curtain. The experimenter remained behind the curtain and out of the infants’ line of sight for the duration of the experiment.

#### Stimuli

**Figure 1:**
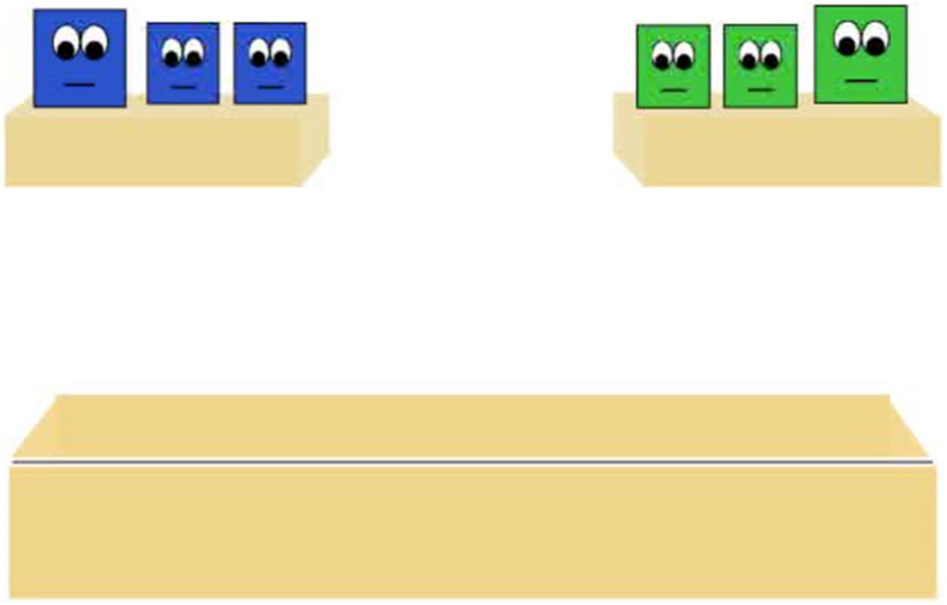
Example of the two groups introduced at the beginning of Experiment 1. Each group consisted of three agents.

Stimuli used were modified from those used in Pun and colleagues^3^, and therefore have been described similarly below. In Experiment 1 of the current work, each group had the same number of agents (3 in each group). Therefore, to equate the number of agents and overall surface area for each group in Experiment 1 (and also maintain consistency between past and present work) we used the same character dimensions from the group of three agents presented in work conducted by Pun and colleagues^3^ (Fig. 1).

Infants were first introduced to two groups taking turns jumping in synchrony (similar to work conducted by Pun and colleagues^3^). Then, two agents from each group jumped behind a barrier, which obscured the agents’ vision. Infants then watched a series of goal *Familiarization* trials (Supplementary Video 1 and2) to demonstrate that one agent from each group had the goal of crossing the platform. Infants saw one agent cross the platform a minimum of two times. This sequence repeated until infants looked away for two consecutive seconds, or had watched four trials in its entirety. Then, infants saw the other agent cross the platform a minimum of two times. This sequence repeated until infants looked away for two consecutive seconds, or had watched four trials in its entirety. Next, infants saw an *Inter-trial* (Supplementary Video 3) that established a conflict between the two agents when they both attempted to complete their goal of crossing the platform at the same time. Importantly, only one group was able to see the conflict, as the other group members’ eyes were obscured by an opaque barrier. Following the *inter-trial,* infants saw a total of two test trials. The test trials began with the same actions depicted in the *inter-trial*. In one trial, infants watched an agent that had social allies witnessing the conflict prevail (Expected outcome) (Supplementary Video 4). In the other trial, infants watched an agent that did *not* have any social allies witnessing the conflict prevail (Unexpected Outcome”) (Supplementary Video 5). After the infant viewed the test event, the animation froze and a static image of the characters remained. Infants’ looking times were measured to the static outcomes of the test event until the infant looked away for two consecutive seconds, or until 30 s had elapsed. The order of these two test trials were counterbalanced across participants. Only infants that viewed both the conflict and the critical sequence in which one of the agents bowed down and moved out of the other agents’ path of motion were included in the final sample^3,22^.

#### Coding

Coders used the computer application *jHaB* (Casstevens, 2007) to record the duration of infants’ looking times^3,22^. For all experiments, infants’ looking times were first recorded by a primary online coder. Data from the primary coder was used in the results. A naive secondary offline coder re-coded 50% of the videos for Experiment 1. For videos that were re-coded, looking times between the two coders were correlated *r* = 0.98 across all trials. Note: the coding methods, procedure and reporting for this experiment is consistent with studies conducted by Pun and colleagues^3,22^, and therefore has been described similarly above.

## Results and discussion

An Analysis of Variance (ANOVA) was run. The dependent variable entered was a difference score between infants’ looking times to the test trials (Unexpected Outcome minus Expected Outcome). Two between subjects factors were entered: gender and trial order (Unexpected Outcome first vs. Expected Outcome first). There was no main effect of gender (F_1,47_ = 0.14, *p* = 0.71), trial order (F_1,47_ = 1.60, *p* = 0.21) or interaction between gender and trial order (F_1,47_ = 0.045 *p* = 0.83). We also ran the same analyses entering age as a covariate. These analyses revealed no significant differences due to age (F_1,47_ = 0.001, *p* = 0.98). Please see Supplementary Information for analyses performed with log-transformed data, which reveal similar results.

**Figure 2:**
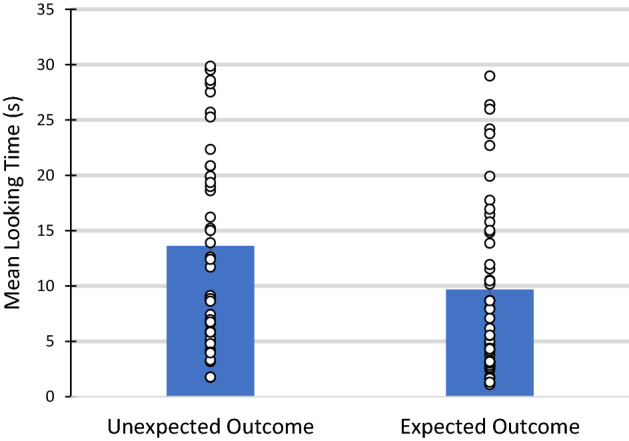
Mean looking time to Unexpected Outcome trial (allies unable to witness conflict) compared with the Expected Outcome trial (allies able to witness conflict) for 9–12 month old infants (within-subjects). Individual data points are depicted. Error bars denote SE of the mean.

Planned comparisons comparing the mean looking times to each trial type (Unexpected Outcome vs. Expected Outcome) revealed that 9–12-month-old-infants looked significantly longer to the Unexpected Outcome trial, in which an agent whose allies were unable to witness the conflict prevailed (*M* = 13.62 s, *SD* = 9.04), compared with the Expected Outcome trial, in which an agent whose allies were able to witness the conflict prevailed (*M* = 9.68 s, *SD* = 7.81), 95% CI [1.31, 6.57], t(47) = 3.02, *p* = 0.004, *d* = 0.47 (Fig. 2).

Our main finding was further supported when the data were examined nonparametrically. Out of 48 participants, 36 infants (75% of the sample) looked longer to the Unexpected Outcome trial in comparison to the Expected Outcome trial, *χ2*_(1,47)_ = 12.00, *p* < 0.001.

In this experiment, 9–12 month-olds only expected an agent to be socially dominant *if* their allies were able to witness the conflict (even if overall group size was equated). These results suggest that infants could identify the group that witnessed (and therefore had knowledge of) the conflict and used this information to predict which agent would be more socially dominant. This is the first study to demonstrate that infants understand the perspectives of multiple agents based on what they can or cannot see and use this information to predict social outcomes (e.g. social dominance).

## Experiment 2

Even when *overall* group size was equated (3 agents in each group), infants only expected an agent to be socially dominant *if* the social allies from that group could *see* the conflict. In essence, Experiment 1, coupled with past research^3^, demonstrated infants’ expectations of social dominance based on relative numerical group size is a *derived* form of dominance. Importantly, it appears to be contingent on whether allies are *aware* that a group member is engaged in intergroup conflict. However, how robust is this ‘visual awareness’ effect? It is possible that when infants are shown discrepancies between group size (i.e. one group has more members than the other), numerical information will be prioritized over eye cues. Further, it is possible that eye cues are only used when numerical group size is equated (as was the case in Experiment 1). To address this outstanding tension, we conducted a second experiment. Infants were introduced to two groups that differed in numerical size (3 agents vs 2 agents). Then, two identically sized agents from opposing groups engaged in a conflict. However, in contrast to the study by Pun and colleagues^3^, *no* social allies in Experiment 2 were able to witness the conflict, as their vision was obstructed. If infants’ expectations of social dominance is contingent on whether allies are *aware* that a group member is engaged in intergroup conflict, then in this case, two identically sized agents (with no allies that can see the conflict) should be evenly matched. Therefore, neither agent should be considered to be socially dominant. However, if infants do not consider the perspectives of social allies and predict social dominance simply by comparing the number of agents that are in each group overall, then the agent from the numerically larger group should be perceived as socially dominant.

### Method

#### Participants

For Experiment 2, data from 48 infants (mean age = 10.08 mo, range = 8.64 mo–11.76 mo, SD = 28 d, 24 females) were recruited from a university database and informed consent from each participants’ legal guardian was provided. Reports from the legal guardian indicated that our final sample was composed of 44% Caucasian, 34% as East Asian, and 22% that identified as other ethnicity/ethnicities. Consistent with the inclusion implemented by Pun and colleagues^3^ and Thomsen and colleagues^23^, an additional 13 participants were excluded from the sample because they did not watch the screen during the critical sequence in which one of the agents bowed down and moved out of the other agent’s path of motion (n = 5), fussed out (n = 5), or because of sibling or parental interference (n = 3).

#### Procedure

The University of British Columbia and the Behavioral Research Ethics Board approved all methods and experimental protocols (approval no. H10-00147). All research methods and experimental protocols described in this manuscript was conducted in accordance with the University of British Columbia’s Behavioral Research Ethics Board guidelines and regulations (approval no. H10-00147). Procedure was identical to Experiment 1.

#### Stimuli

**Figure 3:**
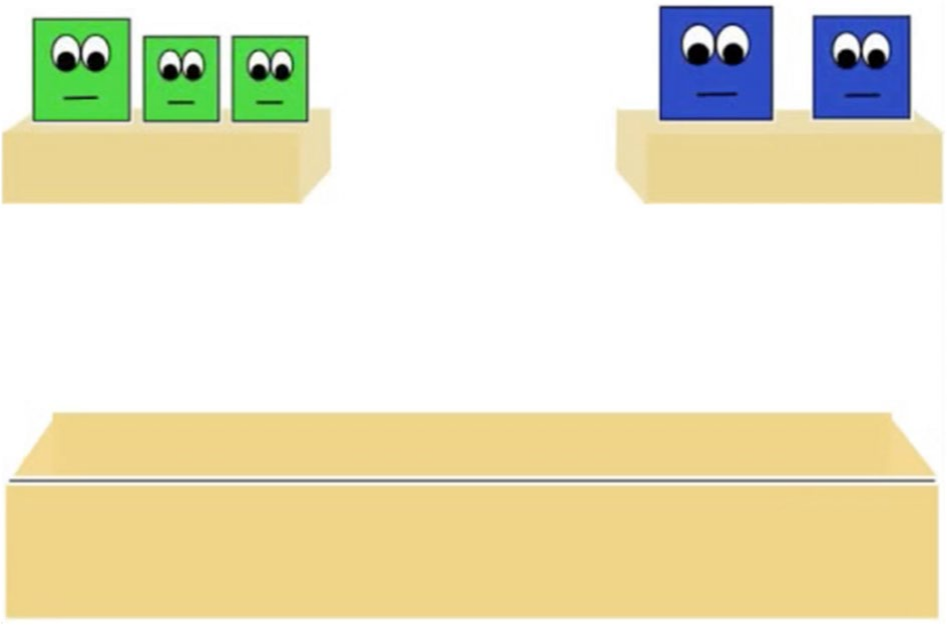
Example of the numerically larger group (3 agents) and numerically smaller group (2 agents) introduced at the beginning of Experiment 2.

Stimuli used were modified from those used in a study conducted by Pun and colleagues^3^, and therefore have been described similarly below. To maintain consistency between the stimuli presented in previous work and our current experiments, the same number of agents and overall surface area that the characters occupied were the same as in the study conducted by Pun and colleagues^3^ (Fig. 3).

Infants were introduced to two groups that differed in numerical group size (3 agents vs 2 agents) and saw each group take turns jumping in synchrony^3^. Then, they watched some of the agents jump behind a barrier (two agents from the larger group, and one from the smaller group), which obscured the agents’ vision. The *Familiarization* trials (Supplementary Video 6 and 7) were then shown to demonstrate that one agent from each group had the goal of crossing the platform. Infants saw one agent cross the platform, a minimum of two times. This sequence repeated until infants looked away for two consecutive seconds, or had watched four trials in its entirety. Then, infants saw the other agent cross the platform a minimum of two times. This sequence repeated until infants looked away for two consecutive seconds, or had watched four trials in its entirety. Next, infants saw an *Inter-trial* (Supplementary Video 8) that established a conflict between the two agents when they both attempted to complete their goal of crossing the platform at the same time. Importantly, *no* social allies were able to see the conflict occur, as their eyes were obscured by an opaque barrier. Following the *intertrial* event, infants viewed two test trials. In one trial, the agent from the numerically smaller group moved out of the way to allow the agent from the numerically larger group could cross the platform (Supplementary Video 9). In the other trial, the agent from the numerically larger group moved out of the way so that the agent from the numerically smaller group could cross the platform (Supplementary Video 10). After the agent crossed the platform, the animation froze and total looking duration for that trial was recorded until the infant looked away for more than two consecutive seconds, or until 30 s had elapsed. The order of these two trials was counterbalanced. After the infant viewed the test event, the animation froze and a static image of the characters remained. Infants’ looking times were measured to the static outcomes of the test trials until the infant looked away for two consecutive seconds, or until 30 s had elapsed. The order of these two test trials were counterbalanced across participants. Only infants that viewed both the conflict and the critical sequence in which one of the agents bowed down and moved out of the other agents’ path of motion were included in the final sample (see^3,22^).

#### Coding

Coders used the computer application *jHaB*^24^ (Casstevens, 2007) to record the duration of infants’ looking times. Infants’ looking times were recorded by a primary online coder. Data from the primary coder was used in the results. A naive secondary offline coder re-coded 50% of the videos for Experiment 2. For videos that were re-coded, looking times between the two coders were correlated *r* = 0.97 across all trials. Note: the coding methods, procedure and reporting for this experiment is consistent with studies conducted by Pun and colleagues^3,22^, and therefore has been described similarly above.

## Results and discussion

An Analysis of Variance (ANOVA) was run. The dependent variable entered was a difference score between infants’ looking times to the test trials (larger group wins minus smaller group wins). Two between subjects factors were entered: gender and trial order (larger group wins first vs. smaller group wins first). There was no main effect of gender (F_1,47_ = 0.029, *p* = 0.87), trial order (F_1,47_ = 2.58, *p* = 0.12), or interaction between gender and trial order (F_1,47_ = 0.013, *p* = 0.91). We also ran the same analyses entering age as a covariate. These analyses revealed no significant differences due to age (F_1,47_ = 1.63, *p* = 0.21). Please see Supplementary Information for analyses performed with log-transformed data, which reveal similar results.

**Figure 4:**
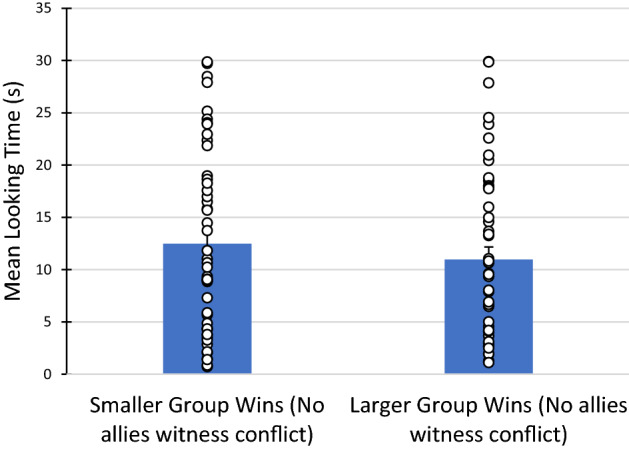
Mean looking time to trial depicting smaller group winning compared to trial depicting larger group winning for 9–12 month old infants (within-subjects). Individual data points are depicted. Error bars denote SE of the mean.

Planned comparisons comparing the mean looking times to each trial type (larger group wins vs smaller group wins) revealed that 9–12-month-olds did not look significantly longer to the trial in which the agent from the numerically larger group prevailed (*M* = 10.96 s, *SD* = 8.35), compared to the trial in which the agent from the numerically smaller group prevailed (*M* = 12.47 s, *SD* = 8.93), 95% CI [1.36, 4.38], t(47) = 1.06, *p* = 0.29 (Fig. 4). This null result is consistent with our original hypotheses, as infants did not expect an agent from either group to be more socially dominant when social allies were unable to witness the conflict. Nevertheless, to provide a more direct test of the null hypothesis, we ran a Bayesian paired-samples t-test^25,26^. The Bayes factor was 5.13, which predicted that infants should look equally long at either outcome trial (when social allies are unaware of the conflict). This provides substantial evidence in favour of the null hypothesis^27^.

These results demonstrate that when social allies did not witness the conflict, infants did *not* expect an agent from a numerically larger group to be socially dominant. This contrasts with previously published findings, which demonstrated that when social allies are *aware* of the conflict, infants expect an agent from a numerically larger group to be socially dominant^3^. Further, these data extend the results of Experiment 1 by demonstrating that infants are sensitive to whether or not social allies have visual access to the conflict when forming judgments about social dominance relationships. Therefore, these data suggest that simply having *more* individuals in one’s overall group does not confer a competitive advantage (if those allies are unable to witness the conflict).

## General discussion

The present research reveals that 9–12 month-old infants are sensitive to whether or not social allies witness intergroup conflict occur, and as a result, this influences expectations of social dominance. In Experiment 1, infants were introduced to two groups. Then, two identically sized agents from opposing groups engaged in a conflict. Although each group had the same number of agents in total (3 in each group), only one group was able to view the conflict at test. The results of Experiment 1 revealed that infants only expected a competing agent to prevail *if* their group had *witnessed* the conflict. In Experiment 2, infants were introduced to a group that had more individuals relative to another group (3 vs 2 agents). When intergroup conflict arose between two identically sized agents, no social allies were able to witness this occur (as their vision was obstructed by a barrier). At test, infants had no expectations about who should prevail. This suggests that infants do not rely on a simple heuristic of ‘she who has more allies will prevail’ (i.e. that the majority will always win over a minority, regardless of whether allies were aware of the conflict). Together, these results suggest that infants are not only capable of using eye cues to determine which agents can *see* the conflict, but are able to ‘take on’ the perspectives of multiple agents to predict the outcome of a dominance contest.

Furthermore, variation in bystander’s physical size (designed to ensure that the overall surface area of each group was equated) did not appear to influence infants’ expectations of social dominance. There may be a number of reasons for this. First, 12–15 month old infants must observe a direct competition between agents, in order to infer the rank ordering of individuals^28^. Since we did not establish rank order within each group in our study, it is unlikely that infants would have been able to infer within-group status differences based on physical size (i.e., no clear leaders vs followers). Second, it is possible that relative differences in physical size may not have been salient enough to predict dominance during group conflict. Third, during intergroup conflict, considerations of within-group size/ rank may be less critical than outnumbering the opposing group. For example, evidence from social species such as lions and chimpanzees reveals that when faced with a conflict, having more allies than the opposing group is the greatest predictor of whether a group will engage in, and win a conflict^2,4,29,30^. Therefore, individual status or physical size may be less predictive of fighting ability when one is able to form a coalition^4,29^.

Taken together, the results of Experiments 1 and 2 demonstrate that infants prioritize information about allies’ awareness of a conflict when making predictions of social dominance. Why might infants predict that the presence of allies that can see (vs. not see) the conflict confers a competitive advantage, even when overall group size is equated? By synthesizing our results with that of^22^, we may generate some plausible hypotheses. Across three experiments, Pun and colleagues^22^ demonstrated that infants as young as 9 months of age use social allegiances to predict how members of social groups should behave during an episode of intergroup conflict. More specifically, infants were introduced to two groups (equal in physical size and number). After viewing a conflict of goals between agents from opposing groups, infants looked significantly longer when an intervening agent helped an outgroup member complete their goal (by pushing their ingroup member off the platform) compared to when an ingroup member indirectly helped their own member complete their goal (by pushing the outgroup member off the platform). This suggests that infants expect an agent to exclusively help an ingroup member during a conflict (even if this requires harming an outgroup member). Therefore, it is possible that infants expect a social group to be socially dominant only *if* their social allies witnessed the conflict *because* these allies are expected to intervene and provide aid during the conflict^22^.

Relatedly, to further explore infants’ sensitivity to the number of allies’ that are able to *witness* the conflict (relative to an opposing group), future work could be conducted. Since our current study (Experiment 1) contrasted a social group that was able to witness the conflict with one that could not, researchers could explore how attune infants are to the relative differences in number of agents that are able to see the conflict occur (vs. are unable to see it occur) when inferring social dominance.

In sum, our study provides novel contributions to the literature by demonstrating that infants are not only capable of using a single agents’ knowledge to predict behavior, but are able to integrate the perspectives of *multiple agents.* Further, to our knowledge this is the first study to demonstrate that manipulating the visual access of social allies (i.e., what they could or could not see through their eyes) influences infants’ third-party evaluations of social groups and expectations of social dominance. Our study provides important confirmation that social dominance is not inferred simply by comparing the number of individuals present, but is contingent upon whether or not social allies are able to witness the conflict.

Crucially, for human infants, attention to eyes is evident at birth^33^ and may have an important role in inferring the mental states of other agents. From an evolutionary perspective, attention to the eyes as a means of communication and signaling may be necessary, and ultimately critical for success when attempting to ambush or hide from prey or rivals^13,34^. Although our study did not specifically track where and how long infants looked at the eyes of the agents, it is an interesting question for future research. For example, future studies employing eye-tracking technology could investigate infants’ attention to the eyes when searching for social allies.

This work, in conjunction with previously published work, suggests that infants within the first year of life understand that the interplay between agents engaged in conflict and social allies/bystanders is dynamic^3,22^. In addition, results from these experiments have provided greater insight into infants’ representation of knowledge (obtained through visual access) and their capacity to reason about the perspectives of social group members during intergroup conflict. More specifically, infants only incorporate agents that can see (and therefore are aware) of a conflict in their predictions of social dominance. This awareness, which appears to emerge in the first year of life, suggests that what others can/ or cannot see through their eyes may be critical to understanding others’ mental states, as it directly impacts what they know, or do not know. Therefore, attention to the eyes may lay the foundations for interpreting and predicting social behaviors^7^.

## Supplementary Information

Supplementary Information 1.

Supplementary Video 1.

Supplementary Video 2.

Supplementary Video 3.

Supplementary Video 4.

Supplementary Video 5.

Supplementary Video 6.

Supplementary Video 7.

Supplementary Video 8.

Supplementary Video 9.

Supplementary Video 10.
