# Supplementary material for: Infants infer third-party social dominance relationships based on visual access to intergroup conflict
Source: Sci Rep. 2022 Oct 29;12:18250. doi: 10.1038/s41598-022-22640-z (PMC9617854; doi:10.1038/s41598-022-22640-z)
Supplement: Supplementary file 1 — Supplementary Information 1. [file 41598_2022_22640_MOESM1_ESM.pdf]

Supplementary Information

Infants infer third-party social dominance relationships based on visual access to intergroup conflict

Anthea Pun*

Susan Birch

Andrew Baron

University of British Columbia

Corresponding author email: antheacp@psych.ubc.ca

This file includes

Supporting Text (Supplementary Results)

Supplementary Video Descriptions

Supporting Text

Supplementary Results

Experiment 1 Additional Analyses

Experiment 1 Results (Log Transformed data)

Looking times from each test trial were first log-transformed and subsequent analyses were conducted on the log-transformed data (see Csibra, Hernik, Mascaro, Tatone & Lengyel, 2016). We ran an ANOVA with a difference score entered as the dependent variable, and entered two between subjects factors: trial order (expected outcome trial first vs. unexpected outcome trial first) and gender. No main effect of trial order was found ($F_{1,47} = 1.24$, $p = .27$). In addition, no main effect of gender ($F_{1,47} = 0.005$, $p = .95$) or interaction between trial order and gender ($F_{1,47} = 0.053$, $p = .82$) was observed. To rule out the possibility of age differences, we ran the same analysis and entered age as a covariate. We found no significant differences due to age ($F_{1,47} = 0.17$, $p = .68$).

Experiment 2 Additional Analyses

Experiment 2 Results (Log Transformed data)

Looking times from each test trial were first log-transformed and subsequent analyses were conducted on the log-transformed data (see Csibra, Hernik, Mascaro, Tatone & Lengyel, 2016). We ran an ANOVA with a difference score entered as the dependent variable, and entered two between subjects factors: trial order and gender. No main effect of trial order was found ($F_{1,47} = 2.15$, $p = .15$). In addition, no main effect of gender ($F_{1,47} = 0.11$, $p = .74$) or interaction between trial order and gender ($F_{1,47} = 0.032$, $p = .86$) was observed. To rule out the possibility of age differences, we ran the same analysis and entered age as a covariate. We found no significant differences due to age ($F_{1,47} = 1.20$, $p = .28$).

Supplementary Video Descriptions

Supplementary Video 1. Familiarization trial. Only bystanders in the blue group can see the blue agent cross. Modified from stimuli published in Pun, Birch & Baron (2016).

Supplementary Video 2. Familiarization trial. Only bystanders in the blue group can see the green agent cross. Modified from stimuli published in Pun, Birch & Baron (2016).

Supplementary Video 3. Inter-trial. Only bystanders in the blue group can see the blue and green agent engage in intergroup conflict. Modified from stimuli published in Pun, Birch & Baron (2016).

Supplementary Video 4. Expected Outcome Test Trial. Only bystanders in the blue group can see the blue agent prevail in the conflict. Modified from stimuli published in Pun, Birch & Baron (2016).

Supplementary Video 5. Unexpected Outcome Test Trial. Only bystanders in the blue group can see the green agent prevail in the conflict. Modified from stimuli published in Pun, Birch & Baron (2016).

Supplementary Video 6. Familiarization trial. No bystanders are able to see the blue agent cross. Modified from stimuli published in Pun, Birch & Baron (2016).

Supplementary Video 7. Familiarization trial. No bystanders are able to see the green agent cross. Modified from stimuli published in Pun, Birch & Baron (2016).

Supplementary Video 8. Inter-trial. No bystanders are able to see the blue and green agent engage in intergroup conflict. Modified from stimuli published in Pun, Birch & Baron (2016).

Supplementary Video 9. Test trial. No bystanders in either group are able to see. Agent from the numerically larger group prevails in the conflict. Modified from stimuli published in Pun, Birch & Baron (2016).

Supplementary Video 10. Test trial. No bystanders in either group are able to see. Agent from the numerically smaller group prevails in the conflict. Modified from stimuli published in Pun, Birch & Baron (2016).
